# Supplementary material for: Safety Messaging Boosts Parental Vaccination Intention for Children Ages 5–11
Source: Vaccines (Basel). 2022 Jul 28;10(8):1205. doi: 10.3390/vaccines10081205 (PMC9413281; doi:10.3390/vaccines10081205)
Supplement: Supplementary file 1 [file vaccines-10-01205-s001.zip › vaccines-1813732-supplementary.pdf]

Supplementary Information

Detailed description of experimental procedures:

We use a survey experiment to do our study. Subjects were hired and screened on MTurk through the CloudResearch Platform. We make our eligibility selection within the part of demographic questions. Eligibility requires:

1. Only parents/guardians of children at ages 5-11 are eligible for this study. If a subject reports that she has no children in this cohort, she is automatically popped out of the experiment and is not regarded as an eligible participant.
2. Given (1) is satisfied, the child (if the subject has only one child at ages 5-11) or at least one of the children (if the subject has multiple children at ages 5-11) has to be unvaccinated for the subject to be an eligible participant.

After screening and demographic questions, they were reminded that they would see a paragraph of text and then randomized into five arms of information. We refer to these arms as "*control*", "*Safety-short*", "*Safety-long*", "*Protect-child*", and "*Protect-family*". After being exposed to these arms, Participants (N_0 = 1770, N = 243) were asked about their intention to vaccinate their children.

Vaccination intention will be measured in two indicators. The first one is a direct question asking for behavioral intentions, assessing the intention to vaccinate their child/children between age 5 and 11 in the coming 30 days. It's measured with a 7-point Likert scale. Another one is a 9-item children-oriented COVID vaccine hesitancy scale resembling Shapiro et al (2018), a vaccination intention scale of children. We adapted it (slightly changed the language) for COVID-19 vaccines in Children ages 5-11.

In the study, all questions are forced responses, meaning that subjects are not allowed to skip questions.

Survey experiment: Parents Vaccination - Info - Final

Start of Block: Intro and Consent

Consent Welcome to our study,

You have been invited to participate in a survey about your attitudes and behaviors during the current COVID-19 pandemic. This study has been approved by the University's Institutional Review Board for Human Subjects.

To participate in this experiment, you need to be at least 18 years old AND a parent or a guardian of at least one child who is between 5 and 11 years old. If you do not match these two conditions, please choose "No, I do not consent".

If you agree to participate in this survey you will be asked about your attitudes and decisions related to health. Most respondents complete the survey in 8-12 minutes. The risks associated with this study are minimal. However, there are some parts of this survey that may cause anxiety or discomfort. It may cause anxiety to think about the coronavirus, and how self and loved ones might be affected by the virus. Furthermore, we will ask about your and your children's general health, which might cause anxiety or sadness when reminded of health issues of self or family members. Participation in this study is voluntary. You may refuse to participate or withdraw at any time.

Please indicate if you consent to take this study. By choosing "Yes, I consent", you agree that you have been provided with a detailed explanation of the procedures to be followed in the project, including an identification of those which are experimental. You understand that: you will be asked to complete a questionnaire concerning my attitudes and opinions on COVID vaccination and to perform tasks on the computer.

If you have further questions about the study, please contact any of the below participants in the research group. Researcher contact information:

Zhihan Cui, Anderson School of Management, UCLA, email: zhihan.cui@anderson.ucla.edu
Sherry Wu, Anderson School of Management, UCLA, email: sherry.wu@anderson.ucla.edu
Lu Liu, Economics - New York University - Shanghai, email: ll4748@nyu.edu
Dan Li, Yale School of Medicine/Yale School of Public Health - dan.li@yale.edu

○ Yes, I consent.

○ No, I do not consent.

End of Block: Intro and Consent

Start of Block: FinishNo

Q129 Sorry, you are not eligible for this experiment. We thank for your time.

○ I understand and finish

End of Block: FinishNo

Start of Block: Basics

Gender Which gender do you identify with?

- ○ Male
- ○ Female
- ○ Others ____
- ○ Choose not to disclose

Age What is your age?

- ○ 18-34
- ○ 35-54
- ○ 55 or over

Party Generally speaking, what's your partisanship identity? i.e., do you usually think of yourself as a Democrat, a Republican, or an Independent?

- ○ Strong Democrat
- ○ Democrat
- ○ Independent Leaning Democrat
- ○ Independent
- ○ Independent Leaning Republican
- ○ Republican
- ○ Strong Republican

Page Break

Age5_11 Are you the parent or guardian of any children between the age of 5 to 11?

- ○ Yes, more than 1 children between 5-11.
- ○ Yes, only one child between 5-11.
- ○ No

Page Break

Display This Question:
If Are you the parent or guardian of any children between the age of 5 to 11? = Yes, only one child between 5-11.

AgeChild What is the age of your child, in years?

- 5
- 6
- 7
- 8
- 9
- 10
- 11

Display This Question:
If Are you the parent or guardian of any children between the age of 5 to 11? = Yes, only one child between 5-11.

VaxChild Has your child already been vaccinated for COVID-19 with at least one shot?

- Yes
- No

Display This Question:
If Are you the parent or guardian of any children between the age of 5 to 11? = Yes, more than 1 children between 5-11.

AgeChildYoung What is the age of your youngest child between 5-11, in years?

- 5
- 6
- 7
- 8
- 9
- 10
- 11

Display This Question:
If Are you the parent or guardian of any children between the age of 5 to 11? = Yes, more than 1 children between 5-11.

VaxYoung Has your youngest child already been vaccinated for COVID-19 at least one shot?

- Yes
- No

Display This Question:
If Are you the parent or guardian of any children between the age of 5 to 11? = Yes, more than 1 children between 5-11.

AgeChildOld What is the age of your eldest child between 5-11, in years?

- ◯ 5
- ◯ 6
- ◯ 7
- ◯ 8
- ◯ 9
- ◯ 10
- ◯ 11

Display This Question:
If Are you the parent or guardian of any children between the age of 5 to 11? = Yes, more than 1 children between 5-11.

VaxOld Has your eldest child already been vaccinated for COVID-19 at least one shot?

- ◯ Yes
- ◯ No

End of Block: Basics

Start of Block: More Demographics

ethnic What is your ethnicity?

- ◯ White
- ◯ Hispanic
- ◯ Black
- ◯ Asian
- ◯ Others ____

---

education What's your highest level of education?

- Some high school or less
- High school graduate
- Completed some college, but no degree
- Associate's degree
- Bachelor's degree
- Master's or professional degree
- Doctorate degree

hhn How many people are there in your household?

- 1
- 2
- 3
- 4
- 5
- 6 or more

Page Break

hhi Your annual household income:

- $25,000 or less
- $25,000 - $35,000
- $35,000 - $45,000
- $45,000 - $55,000
- $55,000 - $70,000
- $70,000 - $85,000
- $85,000 - $100,000
- $100,100 - $120,000
- $120,000 - $140,000
- $140,000 or more

End of Block: More Demographics

Start of Block: Transition

Text

Now, you will be asked to read a piece of text. Please spend at least 30 seconds and read the text carefully.

○ I understand and continue.

End of Block: Transition

Start of Block: Treatment

Q149 Timing
First Click
Last Click
Page Submit
Click Count

Control **If you ignore office culture&protocol as a new employee, you will bring trouble to yourself at work.**

Every office has its own norms of functioning and work practices. The biggest favor you can do yourself as a new employee is to observe and adapt to this routine, instead of following your previous office's schedule or simply doing as you please. Be it the way office communication is carried out, paperwork is handled, workflow takes place, or dress code is followed, you should pay close attention and ask questions, if necessary. If you try to be different or show disregard for the work processes and protocol, you not only risk being ostracized by colleagues but, more importantly, it could harm your career as you may end up offending your boss.

Pay attention to office culture, and you will avoid many problems!

○ I confirm that I have read it carefully and continue.

ProtectChild
If you vaccinate your child for COVID, you will protect his or her health and well-being.

After vaccinated for COVID, your child will be much more unlikely to suffer severe illness or experience short-term or long-term complications. For unvaccinated children, even for those who are lucky enough to recover from COVID in a few days, long-haul symptoms such as irregular heartbeat and memory loss remain threats for their future study and leisure. Besides, if your children have underlying medical problems and are unvaccinated, the chance to get severe symptoms is quite high. Getting your child vaccinated for COVID can also give you and your child the peace of mind to return to more typical activities like in-person schooling, as well as participating in extracurricular activities like playing sports with friends.

Vaccinate your child, and protect his/her health!

○ I confirm that I have read it carefully and continue.

ProtectFamily **If you vaccinate your child for COVID, you will protect yourself and your family members.**

Like adults, children can transmit the coronavirus to others if they're infected, even if they have no symptoms. After your child gets vaccinated, he or she will be unlikely to infect others, which will help prevent or reduce the spread of COVID-19 virus inside your family. When the virus is

unlikely to spread, everyone in your family is protected, not only those who are immune. Getting your child vaccinated for COVID will protect yourself and your vulnerable family members like older relatives or persons with weakened immune systems. Thus, a COVID vaccine shot for your child really helps your family live a safer and more peaceful life.

Vaccinate your child, and protect your whole family!

○ I confirm that I have read it carefully and continue.

SafeShort **Before you vaccinate your child for COVID, it's your right to know that the safety of children's COVID vaccine is effectively guaranteed.**

The COVID-19 vaccine for children has received the most intensive evaluation to ensure its safety and effectiveness, and it's better to get your child vaccinated than never. First, to try to minimize possible risks while obtaining the best immune response, the dosage is specially designed for children ages 5-11. It is a third of the dose that is given in adults and adolescents. Thus, the rate of adverse reactions is even lower than that among adults and adolescents. Moreover, the emergency use of the COVID-19 vaccine for children never means unqualified use, and Phase 1/2/3 Trial in children volunteers were all passed. Outcome measures are powerful to prove the vaccine to be both protective and safe, all reviewed by the external independent Data Monitoring Committee (DMC).

Better safe, than never.

○ I confirm that I have read it carefully and continue.

SafeLong **Before you vaccinate your child for COVID, it's your right to know that the safety of children's COVID vaccine is effectively guaranteed.**

The COVID-19 vaccine for children has received much special and extra attention from many different public organizations and private companies to ensure its safety and effectiveness, apart from it strictly following the standardized procedure of bringing a new vaccine to the public based on U.S. law and regulations.

1. The dosage is specially designed for children ages 5-11: Children aren't little adults, so we can't give them the same vaccine in the same dosage that we gave to adults and older kids. During the COVID-19 vaccine phase 3 trials with children volunteers, different doses were evaluated to find the safest dosage—the dosage that gave the best immune response. Scientists finally settled on a dosage that's a third of that for adults and adolescents. This dosage is very specially and carefully designed to help your children's bodies build immunity to COVID. Some children could feel certain types of body reactions (actually, this rate is lower than that among adults and v because of the specially designed dosage!), but they are mild and will vanish usually within 48 hours. They will cause no long-term damage; instead, they are signs that vaccines are building protection against COVID-19 in your children's body.

2. Emergency use never means unqualified use: Phase 1/2/3 Trial in children volunteers all passed. Numerous researchers have been developing the COVID-19 vaccine since January 2020. The large amount of funding dedicated to vaccine development allowed vaccine candidates to move quickly and thoughtfully through each step of pre-clinical and clinical trials. For example, the Phase 1/2/3 trial of the Pfizer-BioNTech vaccine initially enrolled up to 4,500 children volunteers ages 6 months to under 12 years of age in the United States, Finland, Poland, and Spain from more than 90 clinical trial sites. It was designed to evaluate the safety, tolerability, and immunogenicity on a two-dose schedule (approximately 21 days apart) in three age groups.

Outcome measures are powerful to prove the vaccine to be both protective and safe, all reviewed by the external independent Data Monitoring Committee (DMC).

- I confirm that I have read it carefully and continue.

End of Block: Treatment

Start of Block: Children Vax

Q157 In the U.S., COVID-19 vaccines are now available to children by age group 5 through 11. The U.S. Food and Drug Administration (FDA) has given emergency use authorization to a Pfizer-BioNTech COVID-19 vaccine for this age group since November, 2021. This vaccine involves two injections, given three weeks apart.

Page Break

Display This Question:
If Has your child already been vaccinated for COVID-19 with at least one shot? = No

Vax1_Int
Do you plan to have your child take a COVID-19 vaccination shot within the coming 30 days?

- Definitely yes
- Mostly yes
- Possibly yes
- Might or might not
- Possibly no
- Mostly no
- Definitely no

Display This Question:
If Has your youngest child already been vaccinated for COVID-19 at least one shot? = No

Vax1Young_Int
Do you plan to have your youngest child take a COVID-19 vaccination shot within the coming 30 days?

- Definitely yes
- Mostly yes
- Possibly yes
- Might or might not
- Possibly no
- Mostly no
- Definitely no

Display This Question:
If Has your eldest child already been vaccinated for COVID-19 at least one shot? = No

Vax1Old_Int
Do you plan to have your eldest child take a COVID-19 vaccination shot within the coming 30 days?

- ◯ Definitely yes
- ◯ Mostly yes
- ◯ Possibly yes
- ◯ Might or might no
- ◯ Possibly no
- ◯ Mostly no
- ◯ Definitely no

Page Break

BenefVac To what extent do you agree with the following benefits about COVID-19 vaccines on children from Age 5-11?

	Strongly disagree	Somewhat disagree	Neither agree nor disagree	Somewhat agree	Strongly agree
COVID-19 vaccines are important for my child's health	○	○	○	○	○
Getting a COVID-19 vaccine is a good way to protect my child/children from the disease	○	○	○	○	○
COVID-19 vaccines are effective for children	○	○	○	○	○
Having my child vaccinated is important for the health of others in my community	○	○	○	○	○
Children's COVID-19 vaccines offered by the government program in my community are beneficial	○	○	○	○	○
Please choose "Somewhat disagree"	○	○	○	○	○
The information I receive about COVID-19 vaccines from the vaccine program is reliable and trustworthy	○	○	○	○	○
Generally I do what my doctor or health care provider recommends about COVID-19 vaccines for my child/children	○	○	○	○	○
COVID-19 vaccines carry more risks than influenza vaccines	○	○	○	○	○
I am concerned about serious adverse effects of children's COVID-19 vaccines	○	○	○	○	○

End of Block: Children Vax

Start of Block: Prev

Q136 What's YOUR OWN status of COVID-19 vaccination?

- ○ I am fully vaccinated and had got a booster (3rd shot).
- ○ I am fully vaccinated but had not gotten a booster.
- ○ I am partially vaccinated (with one shot).
- ○ I am not vaccinated.

Display This Question:
If What's YOUR OWN status of COVID-19 vaccination? != I am not vaccinated.

Q137 Have you ever experienced any side effects after your vaccination shots?

- Yes
- No

Display This Question:
If Have you ever experienced any side effects after your vaccination shots? = Yes

Q138 According to your own feelings, how severe was your vaccination side effect experiences?

- Very Severe
- Severe
- Moderate
- Mild
- Very Mild

End of Block: Prev

Start of Block: Infect

Display This Question:
If Are you the parent or guardian of any children between the age of 5 to 11? = Yes, only one child between 5-11.

Infect Has your child ever been tested positive for COVID-19?

- No
- Yes

Display This Question:
If Are you the parent or guardian of any children between the age of 5 to 11? = Yes, only one child between 5-11.

Symptom In the past 14 days, has your child experienced any of the following symptoms? Please choose all that apply.

- ☐ Cough
- ☐ Fever
- ☐ Runny Nose/Nose Congestion
- ☐ Sore throat
- ☐ Fatigue
- ☐ Loss of Smell/Taste
- ☐ Headache
- ☐ None of the Above

Display This Question:
If Are you the parent or guardian of any children between the age of 5 to 11? = Yes, more than 1 children between 5-11.

InfectY Has your youngest child ever been tested positive for COVID-19?

- ○ No
- ○ Yes

Display This Question:
If Are you the parent or guardian of any children between the age of 5 to 11? = Yes, more than 1 children between 5-11.

SymptomY In the past 14 days, has your youngest child experienced any of the following symptoms? Please choose all that apply.

- ▢ Cough
- ▢ Fever
- ▢ Runny Nose/Nose Congestion
- ▢ Sore throat
- ▢ Fatigue
- ▢ Loss of Smell/Taste
- ▢ Headache
- ▢ None of the Above

Display This Question:
If Are you the parent or guardian of any children between the age of 5 to 11? = Yes, more than 1 children between 5-11.

InfectE Has your eldest child ever been tested positive for COVID-19?

- ○ No
- ○ Yes

Display This Question:
If Are you the parent or guardian of any children between the age of 5 to 11? = Yes, more than 1 children between 5-11.

SymptomE In the past 14 days, has your eldest child experienced any of the following symptoms? Please choose all that apply.

- ▢ Cough
- ▢ Fever
- ▢ Runny Nose/Nose Congestion
- ▢ Sore throat
- ▢ Fatigue
- ▢ Loss of Smell/Taste
- ▢ Headache
- ▢ None of the Above

End of Block: Infect

Start of Block: Finish

Finish Thanks so much for your responses! Your secret code is: 8648.

- ○ I understand and finish

End of Block: Finish

Figures

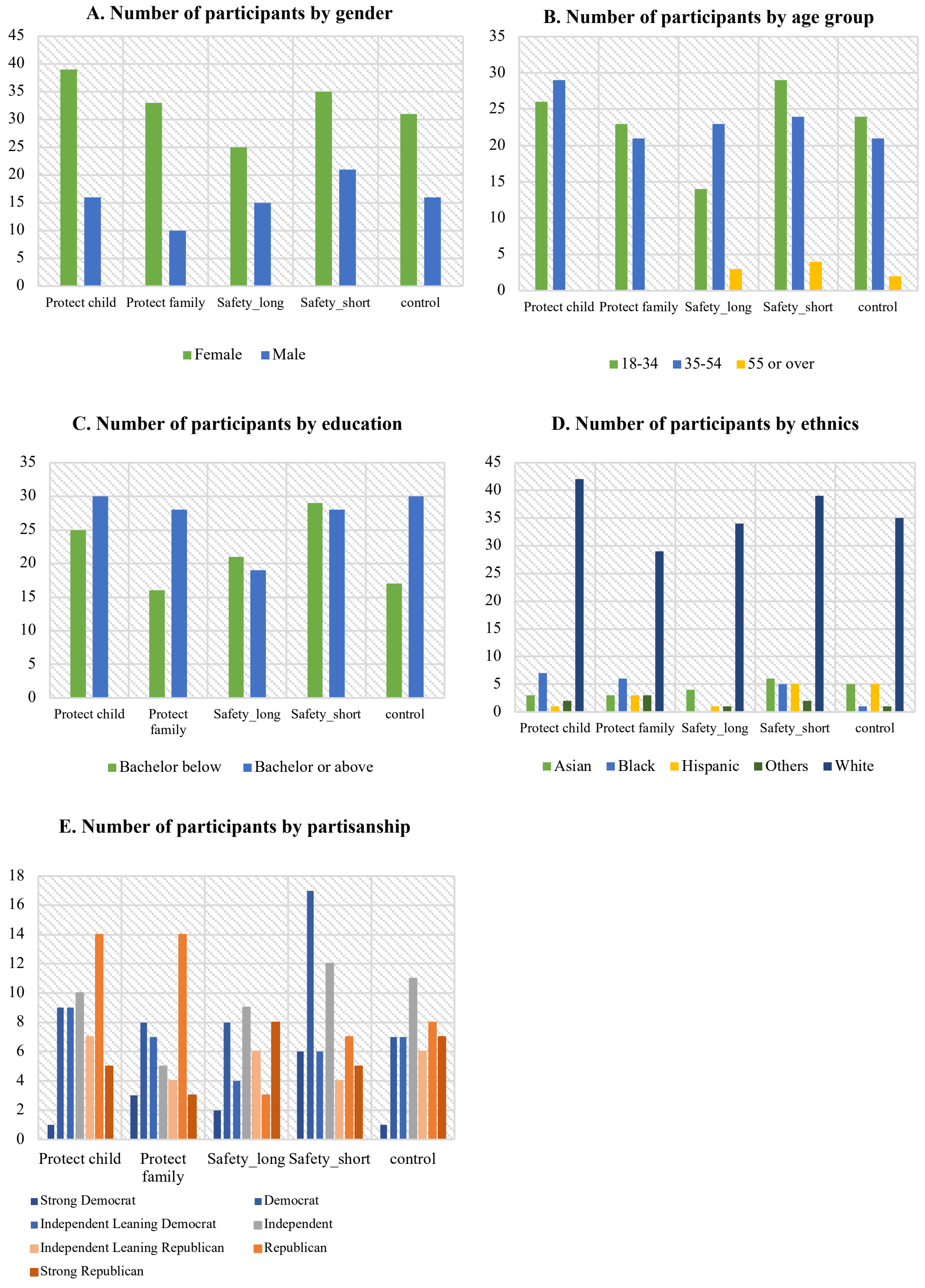

Figure S1. Summary of the Demographics of Different Experimental Groups. Figure S1A – Number of participants by gender; Figure S1B – Number of participants by age group; Figure S1C– Number of participants by education; Figure S1D – Number of participants by ethnics; Figure S1E– Number of participants by partisanship.

Supplementary Analysis:

Factor Analysis of the Children-Oriented COVID Vaccine Hesitancy Scale

Both exploratory and confirmatory factor analyses report a stable two-factor structure that is consistent with the standard vaccine hesitancy scale by Shapiro et al (13). The first factor is captured by Item 1,2,3,4,5,7 and 8 (Item 6 was an attention check), matching the “lack of confidence” factor; and the second factor is captured by Item 9 and 10, matching the “risk” factor. We also analyze the potential asymmetric effects of treatments on these two factors

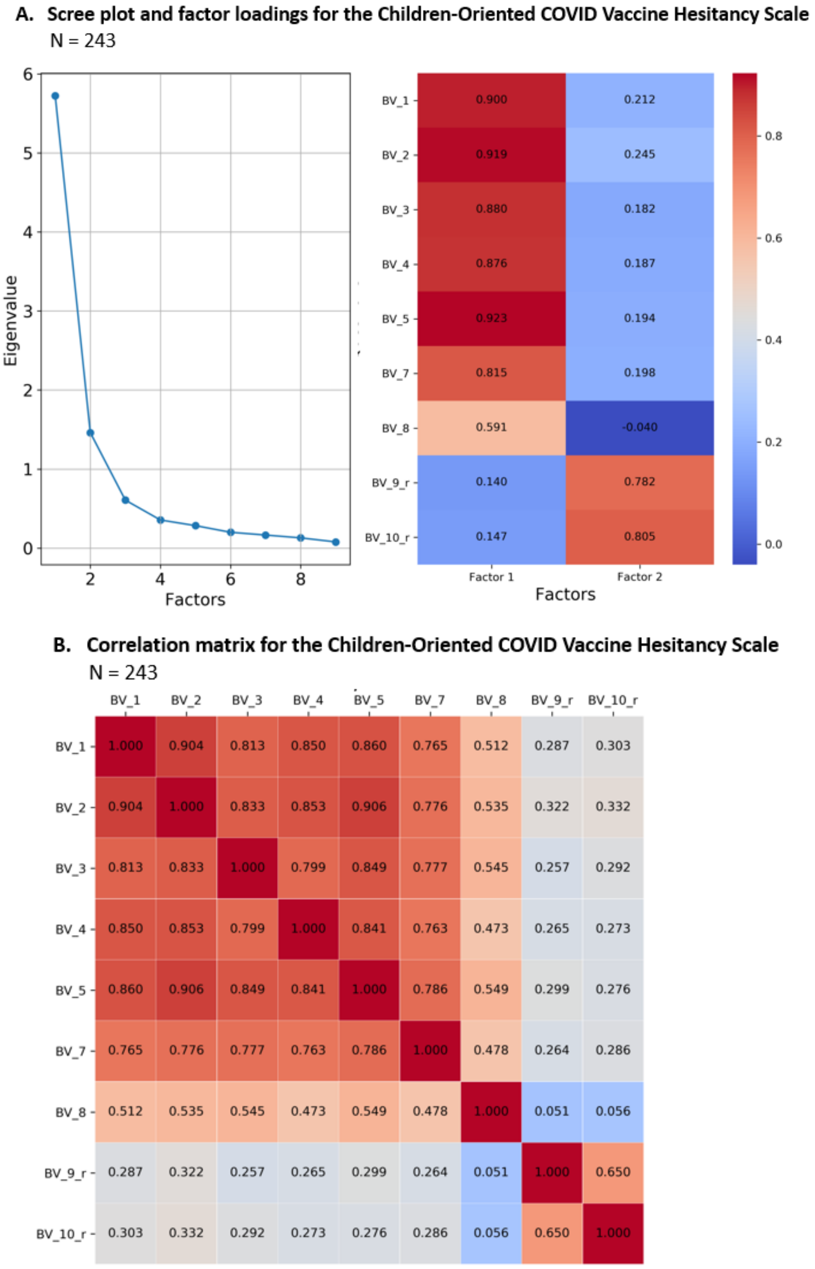

Figure S2. Supplementary Information about the Children-Oriented COVID Vaccine Hesitancy Scale. Figure S2A – Scree plot and factor loadings for the Children Oriented COVID Vaccine Hesitancy Scale. Figure S2B – Correlation matrix for the Children-Oriented Vaccine Hesitancy Scale.

Tables

Table S1. Main Results – Dummy Regression for Vaccination Intention for Treatment Groups

	Parental vaccination intention: per child				Parental vaccination intention: per family	
	OLS	Oprobit	OLS	Oprobit	OLS	Oprobit
Protect child = 1	0.207	0.185	0.154	0.15	0.155	0.152
	(0.50)	(0.84)	(0.36)	(0.60)	(0.38)	(0.70)
Protect family = 1	-0.106	0.0033	-0.294	-0.162	-0.176	-0.0521
	(-0.25)	(0.01)	(-0.66)	(-0.59)	(-0.41)	(-0.22)
Safety_short = 1	1.027**	0.563**	1.022**	0.615**	0.834**	0.469**
	(2.44)	(2.56)	(2.26)	(2.42)	(2.05)	(2.19)
Safety_long = 1	0.163	0.174	-0.106	0.05	0.0928	0.131
	(0.38)	(0.77)	(-0.24)	(0.20)	(0.21)	(0.56)
Republican			-0.870***	-0.565***		
			(-2.90)	(-3.14)		
Female			-0.560**	-0.293		
			(-1.74)	(-1.62)		
Not infected			0.434	0.285		
			(1.40)	(1.51)		
Child age			-0.235***	-0.131***		
			(-3.96)	(-3.70)		
Total family income			0.0807**	0.0449**		
			(2.05)	(2.01)		
Family size			-0.111	-0.0824		
			(-0.78)	(-1.00)		
_cons	1.677***		3.901***		1.745***	
	(6.30)		(4.41)		(5.80)	
N	324	324	291	291	243	243

t statistics in parentheses

* p<0.10, **p<0.05, *** p<0.01

Table S2. Dummy Regression for Vaccination Intention for Treatment Groups: Republican Parents

	Republican_parental vaccination intention: per child			
	OLS	Oprobit	OLS	Oprobit
Protect child = 1	0.17	0.216	0.332	0.333
	(0.31)	(0.61)	(0.56)	(0.78)
Protect family = 1	-0.633	-0.391	-0.626	-0.529
	(-1.57)	(-1.11)	(-1.35)	(-1.23)
Safety_short = 1	2.036***	1.109***	2.443***	1.530***
	(2.81)	(2.74)	(3.75)	(3.43)
Safety_long = 1	0.233	0.208	0.323	0.33
	(0.41)	(0.55)	(0.54)	(0.75)
Female			0.0191	0.0216
			(0.04)	(0.07)
Not infected			0.589	0.531
			(1.40)	(1.33)
Child age			-0.307***	-0.200***
			(-3.92)	(-3.42)
Total family income			0.0861**	0.0485
			(2.12)	(1.48)
Family size			-0.274*	-0.240*
			(-1.83)	(-1.81)
_cons	1.100***		3.413***	
	(3.11)		(2.83)	
N	137	137	122	122

t statistics in parentheses

* p<0.10, **p<0.05, *** p<0.01

Table S3. Dummy Regression for Vaccination Intention for Treatment Groups: Democratic Parents

	Democrat_parental vaccination intention: per child			
	OLS	Oprobit	OLS	Oprobit
Protect child = 1	-0.119	0.00122	-0.841	-0.43
	(-0.16)	0.00	(-0.97)	(-0.99)
Protect family = 1	0.326	0.213	-0.0735	-0.0131
	(0.43)	(0.59)	(-0.09)	(-0.03)
Safety_short = 1	0.265	0.17	0.191	0.159
	(0.38)	(0.49)	(0.27)	(0.42)
Safety_long = 1	-0.7	-0.313	-0.89	-0.47
	(-0.96)	(-0.89)	(-1.06)	(-1.01)
Female			-0.612	-0.265
			(-1.27)	(-1.07)
Not infected			0.435	0.223
			(0.89)	(0.89)
Child age			-0.182*	-0.102*
			(-1.71)	(-1.71)
Total family income			0.0932	0.0624
			(1.08)	(1.46)
Family size			-0.158	-0.092
			(-0.57)	(-0.64)
_cons	2.600***		4.300***	
	(4.97)		(2.76)	
N	131	131	123	123

t statistics in parentheses

* p<0.10, **p<0.05, *** p<0.01

Table S4. Dummy Regression for Parental vaccination hesitancy scale – Full Sample

	Parental vaccination hesitancy scale		
	Overall	Factor 1: benefit	Factor 2: harm
Protect child = 1	0.169	0.0721	0.506**
	(0.82)	(0.300)	(2.340)
Protect family = 1	0.0242	-0.0675	0.345
	(0.11)	(-0.27)	(1.510)
Safety_short = 1	0.538***	0.524**	0.589***
	(2.65)	(2.230)	(2.740)
Safety_long = 1	0.14	0.0494	0.456*
	(0.630)	(0.190)	(1.950)
_cons	2.577***	2.772***	1.894***
	(17.160)	(15.920)	(11.920)
N	243	243	243

t statistics in parentheses

* p<0.10, **p<0.05, *** p<0.01

Table S5. Mediation Analysis: Treatment, Hesitancy and Vaccination Intention

Source	SS	df	MS			
				Number of obs	=	243
				F(4, 238)	=	2.19
Model	10.412548	4	2.603137	Prob > F	=	0.0704
Residual	282.494859	238	1.1869532	R-squared	=	0.0355
				Adj R-squared	=	0.0193
Total	292.907407	242	1.2103612	Root MSE	=	1.0895

	Coef.	Std. Err.	t	P>|t|	[95% Conf.	Interval]
safeshort	0.5888391	0.214658	2.74	0.007	0.1659668	1.011711
protectchild	0.506383	0.2164146	2.34	0.02	0.0800502	0.9327158
protectfamily	0.3450193	0.22854	1.51	0.132	-0.1052	0.7952389
safelong	0.456383	0.2343676	1.95	0.053	-0.005317	0.9180827
_cons	1.893617	0.1589161	11.92	0	1.580555	2.206679

Source	SS	df	MS			
				Number of obs	=	243
				F(5, 237)	=	6.28
Model	121.976019	5	24.395204	Prob > F	=	0
Residual	921.112459	237	3.8865505	R-squared	=	0.1169
				Adj R-squared	=	0.0983
Total	1043.08848	242	4.310283	Root MSE	=	1.9714

vax_int	Coef.	Std. Err.	t	P>|t|	[95% Conf.	Interval]
safeshort	0.5011517	0.3945225	1.27	0.205	-0.276067	1.27837
benefminus	0.5657145	0.1172943	4.82	0	0.3346419	0.7967871
protectchild	-0.1311491	0.3960871	-0.33	0.741	-0.91145	0.649152
protectfamily	-0.3716815	0.4155249	-0.89	0.372	-1.190276	0.4469126
safelong	-0.1653633	0.4274598	-0.39	0.699	-1.007469	0.6767428
_cons	1.673434	0.3633533	4.61	0	0.9576195	2.389249

Effect	Mean	[95% Conf.	Interval]
ACME1	0.3323067	0.0764277	0.6318223
ACME0	0.3323067	0.0764277	0.6318223
Direct Effect 1	0.4918339	-0.28445	1.225322
Direct Effect 0	0.4918339	-0.28445	1.225322
Total Effect	0.8241406	-0.196105	1.805145

Table S6. Interaction Analysis: Treatment and Partisanship

	Parental vaccination intention: per child	
	OLS	OLS
Protect child = 1	0.22	-0.0346
	(0.39)	(-0.06)
Protect child = 1 × Republican =1	-0.05	0.381
	(-0.06)	(0.46)
Protect family = 1	0.357	0.0468
	(0.59)	(0.07)
Protect family = 1 × Republican =1	-0.99	-0.637
	(-1.37)	(-0.75)
Safety_short = 1	0.291	0.338
	(0.56)	(0.60)
Safety_short = 1 × Republican =1	1.745*	2.054**
	(1.96)	(2.27)
Safety_long = 1	-0.0938	-0.373
	(-0.16)	(-0.60)
Safety_long = 1 × Republican =1	0.327	0.626
	(0.40)	(0.71)
Republican	-1.119**	-1.343**
	(-2.20)	(-2.21)
Female		-0.548*
		(-1.72)
Not infected		0.33
		(1.09)
Child age		-0.222***
		(-3.95)
Total family income		0.0830**
		(2.10)
Family size		-0.141
		(-1.03)
_cons	2.219***	4.202***
	(6.03)	(4.74)
N	324	291

t statistics in parentheses

* p<0.10, **p<0.05, *** p<0.01
